# Digital Technology-Based Telemedicine for the COVID-19 Pandemic

**DOI:** 10.3389/fmed.2021.646506

**Published:** 2021-07-06

**Authors:** Yu-Ting Shen, Liang Chen, Wen-Wen Yue, Hui-Xiong Xu

**Affiliations:** ^1^Department of Medical Ultrasound, Shanghai Tenth People's Hospital, Ultrasound Research and Education Institute, Tongji University Cancer Center, Shanghai Engineering Research Center of Ultrasound Diagnosis and Treatment, Tongji University School of Medicine, Shanghai, China; ^2^Department of Gastroenterology, Shanghai Tenth People's Hospital, Shanghai, China

**Keywords:** COVID-19, SARS-CoV-2, telemedicine, respiratory diseases, infectious diseases

## Abstract

In the year 2020, the coronavirus disease 2019 (COVID-19) crisis intersected with the development and maturation of several digital technologies including the internet of things (IoT) with next-generation 5G networks, artificial intelligence (AI) that uses deep learning, big data analytics, and blockchain and robotic technology, which has resulted in an unprecedented opportunity for the progress of telemedicine. Digital technology-based telemedicine platform has currently been established in many countries, incorporated into clinical workflow with four modes, including “many to one” mode, “one to many” mode, “consultation” mode, and “practical operation” mode, and has shown to be feasible, effective, and efficient in sharing epidemiological data, enabling direct interactions among healthcare providers or patients across distance, minimizing the risk of disease infection, improving the quality of patient care, and preserving healthcare resources. In this state-of-the-art review, we gain insight into the potential benefits of demonstrating telemedicine in the context of a huge health crisis by summarizing the literature related to the use of digital technologies in telemedicine applications. We also outline several new strategies for supporting the use of telemedicine at scale.

## Introduction

During the year 2020, the whole world is suffering from a serious health crisis associated with the outbreak of coronavirus disease 2019 (COVID-19), a novel respiratory infectious disease caused by severe acute respiratory syndrome coronavirus 2 (SARS-CoV-2) (1, 2). And as of December 26, 2020, COVID-19 had spread to more than 200 countries worldwide and has affected over 80 million individuals, with nearly 1.7 million known deaths (https://www.worldometers.info/coronavirus/). With the rapid spread of this pandemic, the global healthcare system has been experiencing a severe scarcity of necessary equipment, consumables, and staff. Therefore, hospitals should make efforts to determine the best way to provide timely and high-quality patient care and simultaneously protect providers who are already at the highest risk for contracting this disease (3). Significantly, although tackling the direct impact from COVID-19 is important, in many healthcare settings, it is also vital to maintain the core and critical clinical service. However, in the context of the current situation, the initial reaction of healthcare facilities in a great number of countries is to reduce or even cease many clinical services, such as closure of clinics and postponement of elective surgeries or medical appointments. Actually, such strategies cannot be sustained indefinitely in case this pandemic extends over 6 months, or it must be fully considered if this pandemic even worsens. Therefore, all the healthcare organizations have been continuously striking a balance in their own ways, recognizing that constraints in certain resources can sometimes be limiting, while always placing the safety and care of all the potential patients as their top priority.

As the saying goes, “a crisis provides an opportunity”; the crisis of 2020 would provide an unprecedented opportunity for the progress of telemedicine. During the COVID-19 pandemic, because of the containment efforts such as social distancing, quarantine, and cordon sanitaire, if indicated, medical professionals are confronted with great challenges in delivering healthcare. For such a situation, telemedicine has currently been catapulted into the core role of essential services for patients to help reduce the burden of COVID-19 and preserve some valuable equipment and supplies. Although few kinds of telemedicine services were available during the epidemic of SARS in 2003, since then, they have become more available following the popularization of internet services, the emergence of smartphones, and the fourth-/fifth-generation (4G/5G) transmission technology, making it ideal for addressing some unique challenges posed by this global infectious disease outbreak. For example, implementing telemedicine platforms can mitigate overcrowding in the emergency departments, providing the guidance that patients are seeking while addressing disease exposure concerns of low-acuity patients. Telemedicine can also help to deal with the ongoing healthcare needs from chronic illnesses patients while reducing in-person clinic visits. Besides, telemedicine can deliver specialty care to patients at distant regions where scarce intensivists are available, ensuring that high-quality medical supplies are reserved for those who are in need while reducing human exposures (among both healthcare providers and patients) to this highly contagious disease.

The year 2020 should be the beginning of an exciting decade for telemedicine, particularly when combined with the development and maturation of several emerging technologies, including the internet of things (IoT), 5G networks (4, 5), artificial intelligence (AI) (6, 7), big data analytics (8), and robot and blockchain technology (9), which can be synthetically applied to tackle certain major clinical problems or diseases, such as COVID-19. The emergence of SARS-CoV-2 and its subsequent spread has surpassed those of its predecessors (e.g., SARS), causing an evolving global health crisis. Fortunately, telemedicine networks have currently been established in many countries, incorporated into clinical workflow during this outbreak and shown to be feasible, effective, and efficient in sharing epidemiological data, enabling direct interactions among healthcare providers or patients across distance, minimizing the risk of SARS-CoV-2 infection, and improving access to patient care. Although several reviews have examined the historical use and effects of telemedicine (10–12), however, to our knowledge, the current status of telemedicine approaches based on the ever-emerging technologies, particularly associated with the COVID-19 pandemic, has not been timely summarized yet. Given the excellent performance and growing awareness in digital technology-based telemedicine during the COVID-19 outbreak, the time seems optimal to present a systematic review on telemedicine associated with COVID-19 for healthcare providers and certain biomedical researchers that involves a general understanding of telemedicine systems and the related advanced digital technologies, as well as their applications during the COVID-19 outbreak, aiming to provide structure and guidance for practitioners and certain policymakers to understand various key aspects of this rapidly advancing field and extensively accelerate the application of telemedicine technique in this pandemic, eventually improving the quality of patient care while simultaneously protecting the personal health of medical staffs and saving public resources.

**Figure 1:**
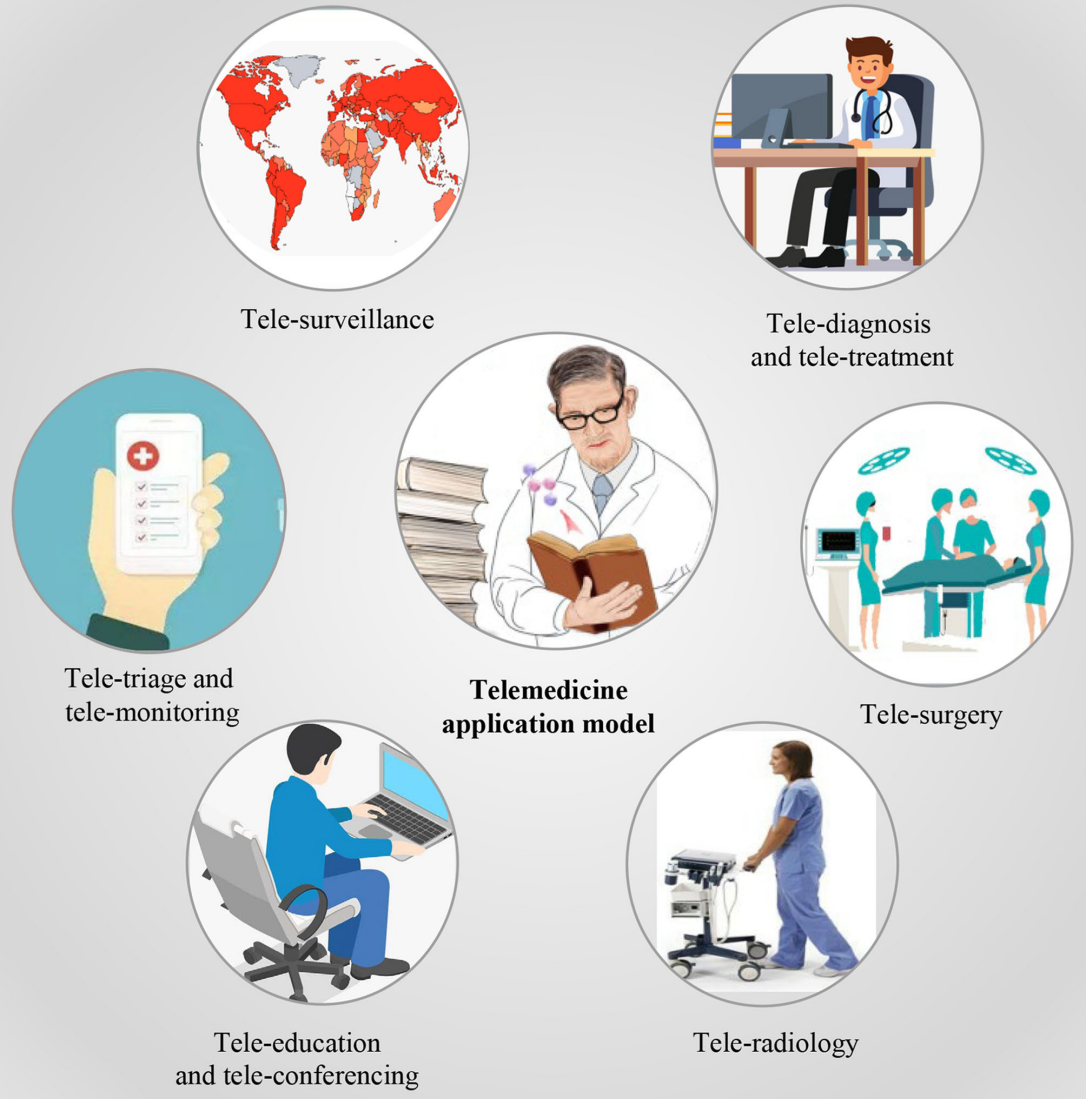
Telemedicine application model in context of the coronavirus disease 2019 outbreak.

In this review, first, we will provide a general understanding of telemedicine as well as several associated technologies. We then review the growing applications of telemedicine within the context of the COVID-19 outbreak by covering tele-consulting, tele-monitoring, telecare, and tele-education (Figure 1). The COVID-19 pandemic can make a good paradigm for presenting the potential benefits of telemedicine nowadays, owing to the emergence of various technological platforms. Finally, we offer our perspective on the potential opportunities and challenges for telemedicine systems in the clinical world, as well as outline several new strategies for supporting the use of telemedicine at scale.

## Telemedicine

Rather than a very specific term, telemedicine can be broadly defined as “the use of communication and information technologies to provide healthcare services without barriers of time and space” (13). As a communication system, telemedicine proposes a brand new model for the production, transmission, and control of medical data and services. And telemedicine has already constituted an organizational innovation, continuously changing the form of diagnostic process, consultation, supervision, and education and training. The use of telemedicine can be traced to as early as the year 1877, when the first telephone exchange system was built in adjoining areas by 21 doctors to allow easier communication with the local drugstore (12). After that, although numerous attempts were made to advance information-based technologies to augment healthcare delivery, the number of clinical telemedicine application practice was still relatively small and restricted, rather than deployed nationwide for quite a long time, mainly due to the clinical, financial, or technical barriers.

**Figure 2:**
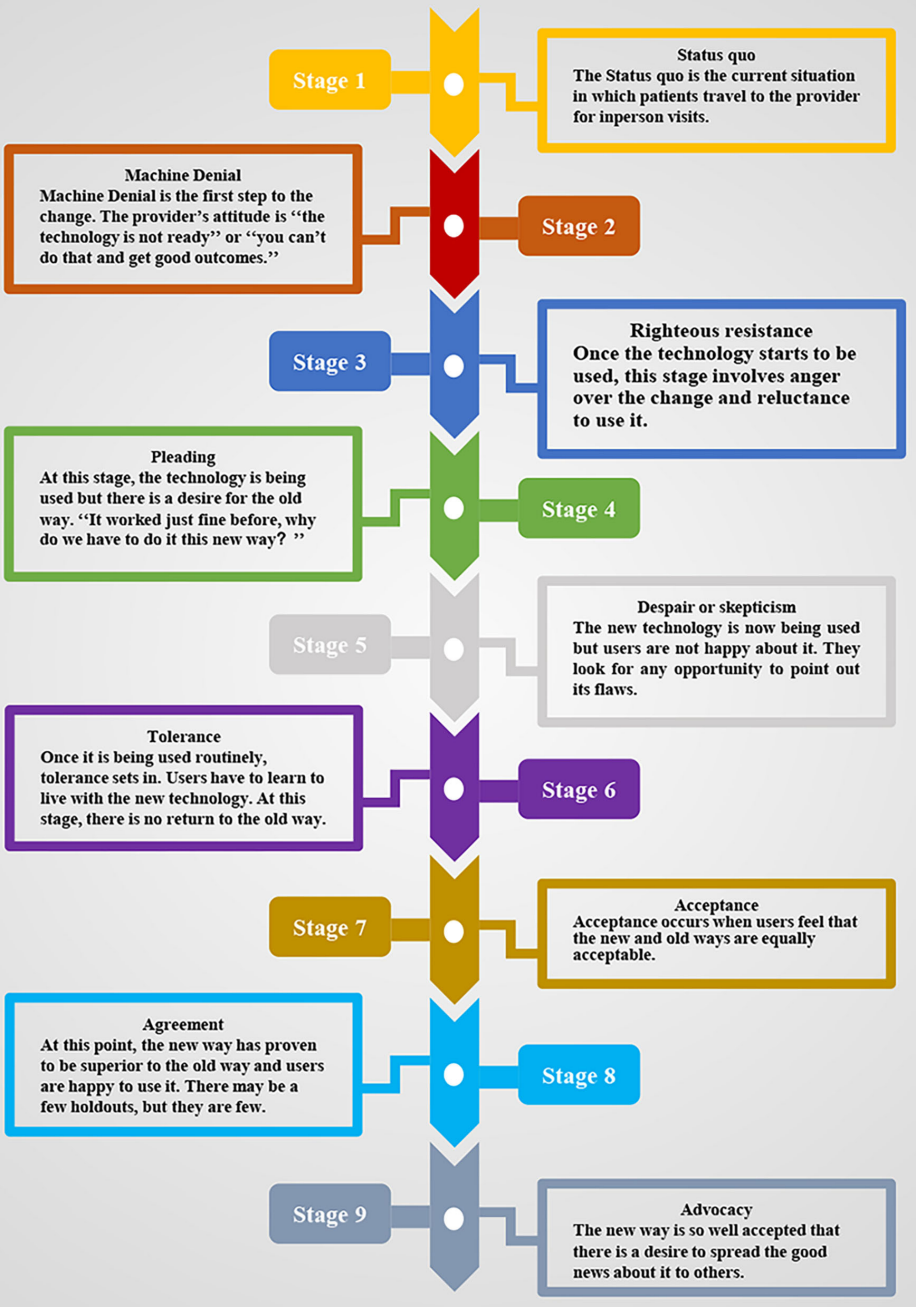
The nine stages of transformational change of telemedicine (19).

Nowadays, telemedicine is widely used across various medical specialties including psychiatry (14, 15), ophthalmology (16), dermatology (17), and neurology (18) and has the potential to impel a transformational change in the way healthcare is delivered via altering the interactive process between patient and provider. Fortunately, it has already undergone five of the nine steps of transformational change presented by Tipton (19) (Figure 2) and is moving beyond the tolerance stage toward the acceptance stage by most healthcare organizations. Once this technology has passed the tolerance stage, it would be difficult to get back to the original way of care, restricting to some episodic in-person visits. We believe that this COVID-19 global outbreak will inevitably accelerate this process and anticipate rapid movement toward the stage of agreement and ultimately expect advocacy to be spread worldwide (20–23).

## Telemedicine-Related Technologies and Their Critical Roles During the COVID-19 Outbreak

Advancements in modern technologies such as IoT, AI, blockchain, and big data have revolutionized the medicine practice to its current state. Especially, 5G-based cloud servers could offer more stable and faster data transmission (up to 100-fold than predecessors) while reducing the latency to 1 ms, enabling them to rapidly and reliably disseminate great amounts of interactive data, from or to anywhere in the world (24–26). Utilizing these ever-growing technologies for telemedicine can provide an entirely immersive experience for those healthcare providers around the world and simultaneously eliminate the perception of distance, marking an optimal transformation from an in-person clinical visit to a synchronized/unsynchronized virtual reality. Some of these technologies and their critical role during the COVID-19 outbreak can be briefly explained as follows.

### Internet of Things

The term IoT can be defined as a worldwide network of the interconnected objects, which are uniquely addressable by standard communication protocols (27), in that all the physical devices, such as smart appliances, autonomous transportation systems, and personal health monitors, which are embedded in the digital technologies, can be networked together to allow these devices to interact with each other via communicating data (27, 28). During the COVID-19 outbreak, the value and function of IoT can be reflected in providing a data platform that could allow public health agencies access to continuously monitor the status of the pandemic. For example, the “Worldometer” presents a real-time update on the exact number of people infected with COVID-19 worldwide, including the daily new cases and severity of disease, as well as disease distribution by countries (https://www.worldometers.info/coronavirus/). Also, Johns Hopkins University has developed a real-time tracking map for following up the COVID-19 patients around the world, by collecting data from the World Health Organization (WHO) and the U.S., European, and Chinese Centers for Disease Control and Prevention (https://gisanddata.maps.arcgis.com/apps/opsdashboard/index.html#/bda7594740fd40299423467b48e9ecf6).

### Artificial Intelligence

AI is a field of computer science that aims to mimic the human perception process to independently represent and interpret complex datasets. This term was first put forward in 1956 as “the conjecture that every aspect of learning or any other feature of intelligence can in principle be so precisely described that a machine can be made to simulate it” (29). Although it was once believed that algorithmic decision making could surpass the accuracy of human judgment, AI was dismissed in 1987 by the *New England Journal of Medicine* due to “the field of medicine is so broad and complex that it is difficult, if not impossible, to capture the relevant information in rules” (30). Since early 2010s, it experienced a resurgence mainly because of the improvements in computing power, development of AI algorithms, and the increasing number and quality of datasets (31). Nowadays, AI is dramatically reshaping the landscape of our lives, ranging from facial recognition and search engines to self-driving cars and natural language processing. AI is rapidly evolving and also plays an important role in this pandemic to improve the detection and diagnosis of COVID-19. For example, based on the large datasets of COVID-19-positive cases from China (https://www.wired.com/story/chinese-hospitals-deploy-ai-helpdiagnose-covid-19/), some AI algorithms have been developed and used as an initial tool for screening suspected cases (i.e., exposure to confirmed cases and travel history to China, South Korea, or Iran); thus, patients at high risk could be isolated or further confirmed with laboratory-based tests. In addition, an AI-based triage system, online medical “chat bot,” (32) has been implemented in context of this outbreak to help people recognize early symptoms and the importance of hand hygiene, potentially alleviating the workload of physicians.

### Big Data

**Figure 3:**
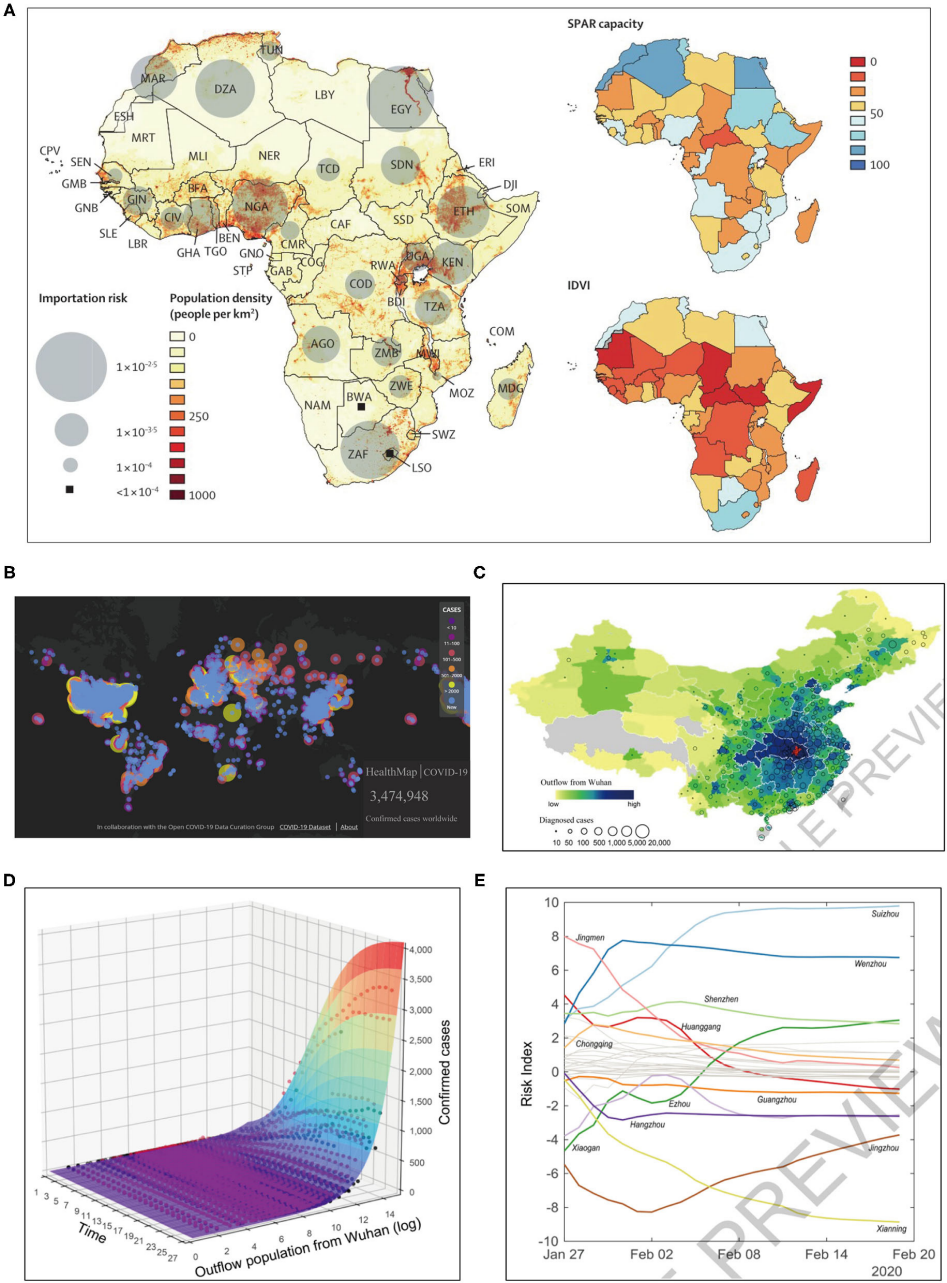
Telemedicine based on artificial intelligence and big data technologies for the surveillance of COVID-19 pandemic. **(A)** Big data based modeling study: preparedness and vulnerability of African countries against importations of COVID-19 (35). **(B)** Online contagious COVID-19 surveillance mapping provided by HealthMap (36). **(C)** Geographic distribution of population outflow from Wuhan through January 24, 2020 (in red) and the confirmed COVID-19 cases in other Chinese prefectures as of February 19, 2020 (37). **(D,E)** Predictive model supported by the population outflow data from Wuhan: **(D)** the surface displays the fitted performance of this epidemiological model, with dots representing actual number of confirmed cases, and **(E)** the risk scores over time present a dynamic picture of the shifting transmission risks in different prefectures (37). SPAR, State Party Self-Assessment Annual Reporting; IDVI, Infectious Disease Vulnerability Index.

Digital phenotyping can yield large datasets that are amenable to the big data analytics. Big data is a term defined by Google that refers to extremely large datasets that can be analyzed computationally to uncover the patterns, trends, and associations, particularly those associated with human interactions and behavior (33). In the field of healthcare, initial forays into the big data have been restricted primarily to analyses of genomics, biomarkers, and clinical information. In the context of this pandemic, big data provides great opportunities for carrying out modeling studies of the viral activity and guiding medical policy organization to enhance the preparation for this outbreak. Based on three global databases from Official Aviation Guide, Location Services of Tencent (Shenzhen, China), and Municipal Transportation Management Bureau of Wuhan, Wu and coworkers conducted a modeled study on “nowcasting” to forecast the COVID-19 disease activity inside and outside China, which could also be used by authorities for public health control worldwide (34). Similarly, utilizing WHO International Health Regulations, Joint External Evaluation reports and the Infectious Disease Vulnerability Index, and State Parties Self-Assessment Annual Reporting Tool, Gilbert et al. (35) evaluated the vulnerability and preparedness of African countries in coping with COVID-19, which would help to raise awareness in the health authorities to better prepare for this viral outbreak in Africa (Figure 3A). Concurrently, it has already been increasingly clear that big data analytics would be quite necessary to detect and further understand the complex genetic sequence and biological mechanisms of SARS-CoV-2 (38).

### Blockchain

Blockchain was first conceptualized and gained recognition in 2009 due to Bitcoin, the first digital cryptocurrency (39). Even if it was at first regarded as a public, decentralized collection of several technologies to allow for storing data permanently while being immune to fraud without the need of a central or reliable authority like a bank, this concept has currently been not confined to the exchange of payments. With numerous investments from venture capital and large multinational technology companies, the use of blockchain technology is growing in markets beyond finance, such as the healthcare industries (40–42). At the hospital level, blockchain is one of the widely used technologies for electronic medical records (EMRs) in the modern hospital ecosystems. And at the patient level, blockchain is increasingly used for sharing health data between patients and providers. Also, blockchain-based telemedicine interventions are enabling remotely monitor patient via biosensors, bridging access gaps regarding patient-level health services (43). Blockchain technology might have a role in addressing two challenges associated with the internet: trust and identity (44). It is capable of digitizing reliable records in a way that is independent of a single database, which would allow the validation of certain clinical credentials across different care providers, providing great opportunity for supporting adoption of a new digital environment for improving healthcare (45). In addition, utilizing blockchain technology in research might have potential for acquiring more public records of database that could help to address the reproducibility challenge via creating links across related communities. Besides, there are also attempts to use blockchain to battle with this COVID-19 outbreak. For example, many hospitals of China—or in some other countries—are collaborating with blockchain pharmacies and companies to deliver the medication to patients' doorsteps, thus allowing hospitals to deliver medications timely and also with accurate tracking (32).

Actually, the above technologies are highly interrelated and ultimately facilitate the running of telemedicine. That is to say, the proliferation of IoT (e.g., instruments and devices) in hospitals and clinics could contribute to the establishment of a greatly interrelated digital ecosystem, enabling real-time large-scale data collection, which can then be used by the AI, especially deep learning system, to fully understand the healthcare trends and the model risk associations and to predict outcomes. This should be enhanced by the blockchain technique, a network of computers distributed in different organizations and a back-linked database with some pre-designated cryptographic protocols, integrating the peer-to-peer networks to allow data to be copied in various physical locations, with certain modified algorithms to ensure the data are not only secured but also traceable (9). The good cooperation between these technologies, together with 5G-based cloud servers, can offer network platforms as well as act as a dependable resource for the currently unprecedented development of telemedicine.

## Overview of the Application of Telemedicine Based on Modern Technologies During the COVID-19 Outbreak

In this article, we have used PubMed or Web of Science databases to summarize the literature based on the keywords “fifth generation (5G) networks” OR “Internet of things” OR “artificial intelligence” OR “block-chain” OR “big data” and “robotics” AND “telemedicine” in the period since the COVID-19 pandemic to the present to augment the performance of traditional public health strategies used in tackling the COVID-19 outbreak, including the (1) surveillance, screening and triage, diagnosis, treatment and monitoring of COVID-19, and (2) mitigation of the impact to healthcare system indirectly related to COVID-19 including management of common and chronic conditions, tele-surgery, tele-psychology, tele-education, and tele-conferencing (Table 1).

**Table 1:** Digital technology based telemedicine and their impact on public-health strategies during the COVID-19 pandemic.

**Digital technology**	**Study**	**Country**	**Intervention**	**Patient location**	**Telemedicine Modality**	**Findings**
IoT, Big data	Worldometer	–	–	At home/In clinic	Surveillance the status of pandemic	Presents a real time update on the exact number of people infected with COVID-19 worldwide, including the daily new cases and severity of disease, as well as disease distribution by countries
IoT, Big data	Johns Hopkins University	The United States	Develop a real time tracking map for following the COVID-19 patients	At home/In clinic	Surveillance the status of pandemic	Following the COVID-19 patients around the world
Big data	Wu et al. (34)	China	Conduct a modeled study on “nowcasting” based on global databases	At home/In clinic	Surveillance the status of pandemic	Forecast the COVID-19 disease activity inside and outside China, which could also be used by authorities for public health control worldwide
Big data	Gilbert et al. (35)	Belgium; France; the United States; Côte d'Ivoire; United Kingdom	Evaluate the vulnerability and preparedness of African countries in coping with COVID-19	At home/In clinic	Surveillance the status of pandemic	Countries with the highest importation risk (i.e., Egypt, Algeria, and South Africa) have moderate to high capacity to respond to outbreaks. Countries at moderate risk (i.e., Nigeria, Ethiopia, Sudan, Angola, Tanzania, Ghana, and Kenya) have variable capacity and high vulnerability. Three clusters of countries that share the same exposure to the risk originating from the provinces of Guangdong, Fujian, and the city of Beijing, respectively.
Big data	HealthMap (46)	The United States	Develop an online surveillance-mapping tool	At home/In clinic	Surveillance the status of pandemic	Surveilling COVID-19 pandemic
Big data	SORMAS (46)	Germany	Develop an online surveillance-mapping tool	At home/In clinic	Surveillance of the COVID-19 pandemic	Surveilling COVID-20 pandemic
Big data	Qin et al. (47)	China	Exploite the big data technique	At home/In clinic	Surveillance the COVID-19 pandemic	By employing techniques such as subset selection method, new COVID-19 suspected and confirmed cases could be detected 6-9 and 10 days in advance, respectively.
Big data, AI	Yang et al. (48)	China	Establish a modified SEIR model	At home/In clinic	Surveillance the COVID-20 pandemic	A 5-day delay in the adoption of stringent public health measures by the Chinese authorities would have leaded to a COVID-19 epidemic size increased by up to three times. Also, loosening or lifting the lock-down intervention in the Hubei province would cause a second peak by mid-March until late April.
Big data, AI	Blue Dot	Canada	Develop an AI-based surveillance system	At home/In clinic	Surveillance the COVID-19 pandemic	Reveal news of the pandemic, which is widely regarded as the first organization to detect the epidemic outbreak in late December, 2019, well ahead of any other international institution and agency.
Big data, AI	Zhang et al. (49)	–	Develop an AI-based surveillance and prediction system	At home/In clinic	Surveillance the COVID-19 pandemic	A real-time system to surveilling and sentiment prediction COVID-19 pandemic.
AI	Zivkovic et al. (50)	–	Improve the current time-series prediction algorithms system	At home/In clinic	Surveillance the COVID-19 pandemic	Achieved good predictive efficacy of COVID-19 pandemic by using a hybrid algorithmic approach that combines machine learning, an enhanced beetle tentacle search population intelligent metaheuristic algorithm.
AI	Yan et al. (51)	China	Develop an “online self-assessment tool” supported by AI	At home	Screening and Triage COVID-19 patients	Help individuals self-evaluate the risk of COVID-19.
AI	Srinivasa Rao et al. (52) Zahedi et al. (53)	the United States	Provided a framework based on AI algorithm that could enable quick identification of COVID-19 cases	At home	Screening and Triage COVID-19 patients	Depending on these replies, this algorithm could send alerts to the respondent, as well as clinics or the mobile health units, for further health visits and case confirmation.
AI	D'Angelo et al. (54)	Italy	Establish a convolutional deep neural network-based human activity classifier	At home/In clinic	Screening and Triage COVID-19 patients	Improve the performance of the new COVID-19 patient tracking application by using a human activity classifier based on convolutional neural networks and multichannel images.
Robot	Global Health Security (55, 56)	China	Develop robots to treat and test Covid-19 patients	At home/In clinic	Screening and Triage COVID-19 patients	Develop robots to treat and test Covid-19 patients in a bid to protect health workers.
Blockchain	Ting et al. (32)	Singapore	Hospitals collaborating with blockchain pharmacies and companies	In clinic	Treatment of COVID-19 patients	Deliver the medication of patients to their doorsteps, thus allowing hospitals deliver medications timely also with accurate tracking.
5G, Blockchain	Hong et al. (57)	China	Conduct CT scanning	In clinic	Diagnosis of COVID-19 patients	The first reported case of remote CT scanning during the COVID-19 pandemic.
AI, Big data	Zhang et al. (58)	China	Reported an AI-powered CT diagnostic system	In clinic	Diagnosis of COVID-19 patients	Diagnose COVID-19 with an accuracy of 92.49%, and had been made available globally to assist the clinicians to combat COVID-19.
5G, Blockchain	Li et al. (59) Lv et al. (60)	China	5G-based teleultrasound network	In clinic	Diagnosis and treatment of COVID-19 patients	Facilitate “on-line” imaging data transmission, and the further “real-time” diagnosis or operation guidance for COVID-19 patients, especially those in ICUs.
5G, Robot	Li et al. (59)	China	5G remote robotic ultrasound diagnostic” system	In clinic	Diagnosis of COVID-19 patients	Enable real-time remote control for ultrasound scanning, thus eliminating exposure to COVID-19 to the greatest extent.
AI	Li et al. (61)	China	AI diagnostic model based on chest CT	In clinic	Diagnosis of COVID-19 patients	Distinguishes COVID-19 from community acquired pneumonia with the sensitivity and specificity of 90% and 96%, with an AUC of 0.96
AI, Big data	CLEW (62)	Israeli	AI-powered tele-ICU system	In clinic	Monitoring status of COVID-19 patients	Support monitoring COVID-19 patients status with certain respiratory deterioration prediction models, which was later installed in two Israeli hospitals.
5G, Robot	Tian et al. (63)	China	5G based tele-robotic spinal surgery	In clinic	Mitigation of the impact to healthcare system indirectly related to COVID-19	All the 12 patients treated with this technology had substantial relief from their symptoms, while without any intraoperative adverse event.
AI	Wang (64)	China	Tree Holes Rescue (a kind of AI psychological programme)	At home	Mitigation of the impact to healthcare system indirectly related to COVID-20	Recognized individuals at risk of suicide by monitoring and analyzing the messages posted on Weibo, and further alerting the designated volunteers to take action accordingly.

*5G, fifth generation; AI, artificial intelligence; IoT, internet of things; CT, computed tomography; US, ultrasound; COVID-19, coronavirus disease 2019; WHO, World Health Organization; ICU, intensive care unit; SEIR, Susceptible-Exposed-Infectious-Removed*.

## Surveillance, Screening and Triage, Diagnosis, Treatment, and Monitoring of COVID-19

### Surveillance and Sharing Data of the COVID-19 Pandemic

Controlling epidemics requires effective and extensive surveillance and timely sharing of epidemiological data (65). During this COVID-19 pandemic, one notable example is Iran (66): as of February 23, 2020, it confirmed its first 43 cases with a fatality rate of 19%, while the reporting transmission modeling prompts that the actual number of infected cases was in the thousands. The rapid global spread of the COVID-19 outbreak has been catalyzed by the insufficient communication and underreporting (67). Healthcare facilities, from the local hospital to WHO, require tools that could improve the speed of communication to better manage the spread of infectious diseases. Telemedicine system based on modern technologies can be implemented for this purpose, as it possesses connectivity, computational power, and hardware to facilitate the electronic reporting and epidemiological databasing. AI and big data can help address the unprecedented amounts of data derived from public health surveillance and real-time epidemic outbreak monitoring (68). For example, the online surveillance mapping tools, including the HealthMap (36) (Figure 3B) and Surveillance Outbreak Response Management and Analysis System (SORMAS) (46), have already been used for surveilling COVID-19, with great potential to improve early detection of the infectious diseases compared with traditional epidemiological tools (69). Sun et al. (70) testified to the strength of monitoring COVID-19 epidemiological information from news media and social networks to help reconstruct the progression of an outbreak and to provide detailed patient-level data during a health emergency. Similarly, Qin et al. (47) exploited the big data technique to predict the number of new infected patients, either suspected or confirmed. Besides, Blue Dot, a Canadian company, has developed an AI-based surveillance system to reveal news of the pandemic, which is widely regarded as the first organization to detect the epidemic outbreak in late December 2019, well ahead of Chinese authorities and any other international institutions and agencies (71, 72). Zhang et al. (49) described a real-time system for sentiment prediction of COVID-19 epidemiological information based on Twitter data. And by evaluation, it was found that predictive analysis with a random forest classifier has the best performance. Zivkovic et al. (50) achieved good predictive efficacy by using a hybrid algorithmic approach that combines machine learning, an enhanced beetle tentacle search population intelligent metaheuristic algorithm, and an adaptive neuro-fuzzy inference system to predict new COVID-19 patient cases.

Considering that sudden large-scale human migration could amplify the localized outbreak into widespread epidemic (73, 74). rapidly and accurately tracking the aggregate population flows can be epidemiologically informative. Jia et al. (37) based on the mobile phone datasets of 11,478,484 people egressing or transiting through the COVID-19 epidemic epicenter, Wuhan of Hubei Province, developed a spatio-temporal “risk source” model (Figures 3C–E) to forecast confirmed cases and also to identify the highly transmission-risk locations at an early stage, which can help the local authorities to make better risk assessment and further to plan allocation of the limited resources before ongoing outbreaks. Similarly, supported by population migration data, Yang et al. (48) established a “Susceptible-Exposed-Infectious-Removed” (SEIR) model, which, combined with an AI approach, is able to predict the pandemic curve, and the authors presented that a 5-day delay in the adoption of stringent public health measures by the Chinese authorities would have led to a COVID-19 epidemic size increased by up to three times. Also, loosening or lifting the lockdown intervention in Hubei Province would cause a second epidemic peak by mid-March until late April. Colubri et al. (75) used AI technique to harmonize several datasets from the Ebola epidemic to offer informed access to the evidence-based guidelines. And then these guidelines were incorporated into an app of the Ebola Care Guidelines (75). Many of these lessons can be re-applied for COVID-19. The app has the potential of including real-time updates of the evidence-based guidelines to inform both the general population and the healthcare providers. Integrating real-time updates into the EMRs could also serve as a reliable resource for guiding clinical practice. Telemedicine platform can be paired with the accessible information technologies to provide real-time, shareable, epidemic information during outbreaks like COVID-19, which empowers individuals, clinicians, and national and global healthcare agencies to adopt coordinated control strategies.

### Screening and Triage of COVID-19 Patients

A central strategy for healthcare pandemic control is “forward triage,” sorting patients before they arrive in the clinical departments. Direct-to-consumer telemedicine system, an approach to forward triage, could allow patients to be timely and efficiently screened; thus, it is patient-centered and simultaneously conducive to the self-quarantine, protecting providers, patients, and the community from disease exposure. Health institutions in many countries are offering free triage telemedicine assessments for COVID-19 by providing patients with access to certain websites or mobile apps that involve a short survey regarding the age, symptoms, and travel history of patient. Based on these results, respondents will be offered with tips, to visit a nearby COVID-19 testing site, or be connected digitally with physicians. In addition, health-focused chat bots, such as Lark Health (76) and Buoy Health (77), can also be used to interpret symptoms of individuals and further present appropriate advices. And Yan et al. (51) provided an “online self-assessment tool” supported by AI technologies to help individuals self-evaluate the risk of COVID-19, thus relieving pressure on the healthcare staff and alleviating the anxiety of patients.

**Figure 4:**
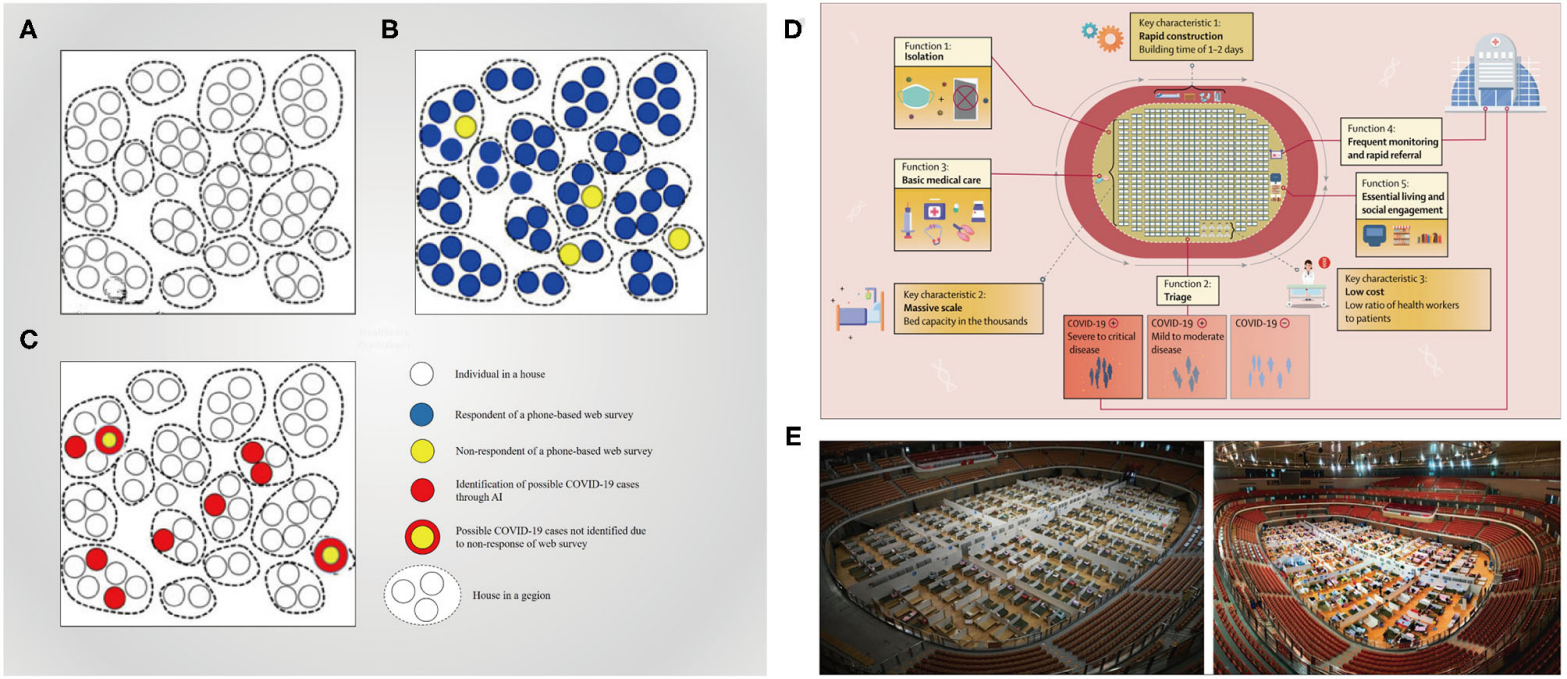
Telemedicine system for the screening and triage COVID-19 patients. **(A–C)** Conceptual framework of collecting data and identifying possible COVID-19 cases (52); a geographic region (e.g., a village, town, county, or city) with households in it **(A)**. The respondents and non-respondents of a phone-based web survey **(B)**. The possible identified COVID-19 cases among the respondents and non-respondents of the survey (78) **(C)**. **(D,E)** Fangcang shelter hospitals equipped with telemedicine system and their key characteristics and essential functions (79).

In response to this pandemic, many countries have devoted to virtual medical care development, which could allow patients and providers to communicate though smartphones or webcam-enabled computers. Community residents with respiratory symptoms, which might be early signs of COVID-19, could consult using the online clinic consultation services. Srinivasa Rao et al. (52) and Zahedi et al. (53) provided frameworks (Figures 4A–C) based on AI algorithm and IoT that could enable quick identification of COVID-19 cases, by performing risk assessment on the symptoms and signs associated with this novel coronavirus though web- and mobile-based survey, respectively. And, depending on the replies, this algorithm could send alerts to the respondent, as well as to clinics or the mobile health units, for further health visits and case confirmation. Automated screening algorithm can be integrated into the tele-consultation process, and the local epidemiologic information can also be utilized to standardize the screening and practice patterns between healthcare providers. More than 50 U.S. health agencies already have such programs. Kaiser Permanente, Jefferson Health, Mount Sinai, and Cleveland Clinic, for example, all leverage telemedicine platform to allow clinicians to screen potential patients who are at home (67). The D'Angelo team (54) utilized a human activity classifier based on convolutional neural networks and multichannel images to improve the performance of the new COVID-19 patient tracking application.

During this pandemic, Fangcang shelter hospitals (Figures 4D,E), which are a novel public health concept built by converting the existing public venues (e.g., stadiums and exhibition centers) into healthcare facilities, were first implemented in China to tackle the COVID-19 outbreak (79). In Wuhan of Hubei Province, they served to isolate patients experiencing mild-to-moderate symptoms of COVID-19 from their families, and importantly, digital technology-based telemedicine was integrated into the workflow of Fangcang shelter hospitals to support medical care; that is, health workers had access to the electronic information systems, which were connected with higher-level hospitals and based on cloud platforms, for record keeping, data transfer, and remote monitoring (80), thus offering high-quality medical care and fulfilling an important triage function. Fangcang shelter hospitals could be powerful components of the national response to COVID-19 and was even considered a major reason for the successful pandemic control in China (79). In addition, the safety of healthcare workers is greatly protected by the use of a remote-controlled robot with pharyngeal swab detection and a robotic arm for SARS-CoV-2 detection to screen suspected patients (55, 56).

### Diagnosis and Treatment of COVID-19 Patients

**Figure 5:**
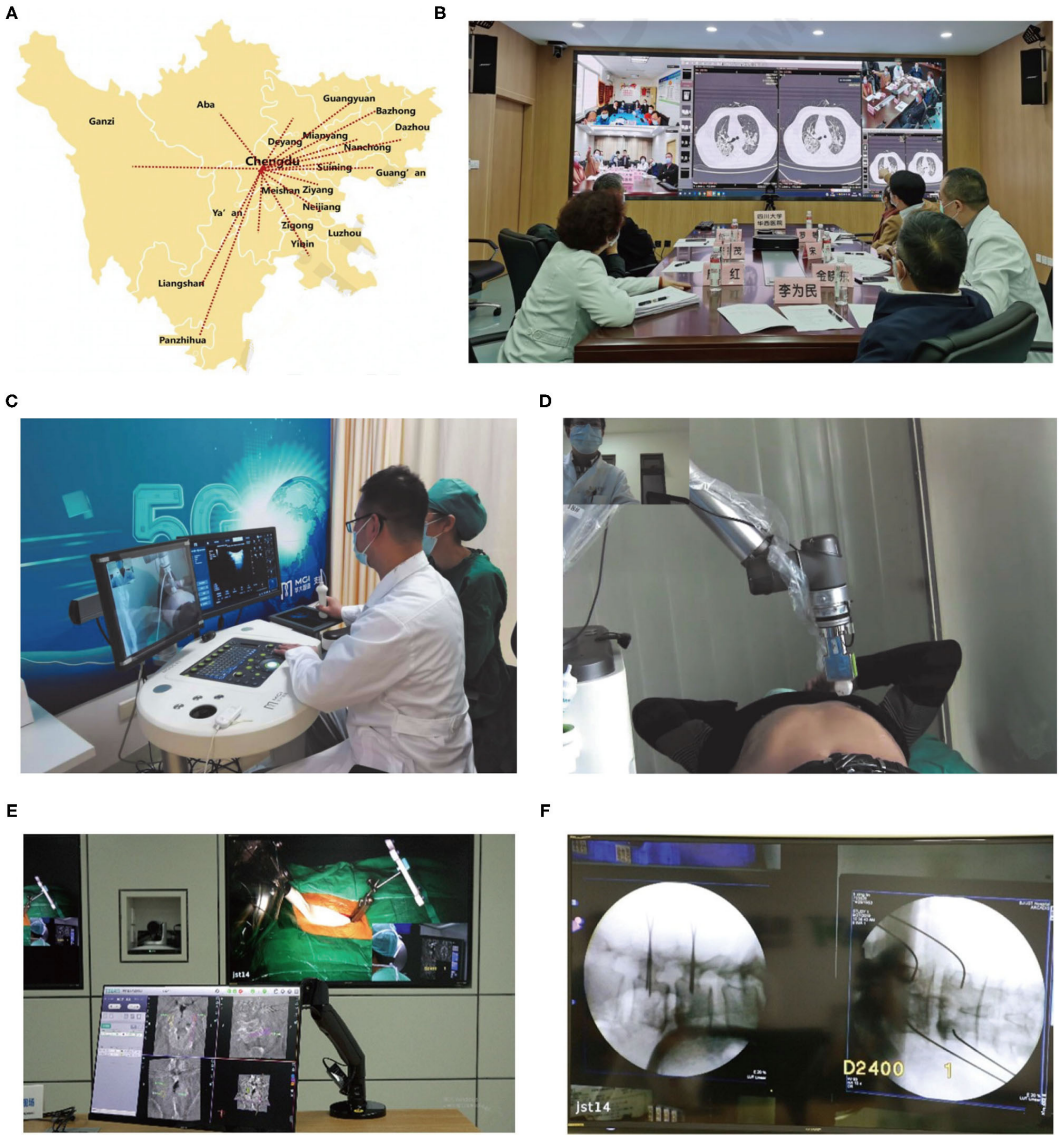
Telemedicine based on fifth-generation (5G) network and robotic technology for disease diagnosis and treatment during the COVID-19 outbreak. **(A)** 5G telemedicine platform of Sichuan Province of China developed during this pandemic. **(B)** The web-based real-time video tele-consultation provided by a multidisciplinary medical team based on “5G Dual Gigabit network” to deal with cases vulnerable to severe COVID-19 in western China (57). **(C,D)** 5G remote robotic ultrasound diagnostic system used in Fangcang shelter hospitals (82). **(E)** 5G network-based tele-ultrasound system tele-robotic spinal surgery including screw planning at master control room and **(F)** K-wire placement (63).

In context of the COVID-19 outbreak, the telemedicine system can provide a good platform for enhancing communication among healthcare providers, could increase diagnostic accuracy of some difficult cases, and improve the treatment results of severe or critical COVID-19 patients in areas with limited medical resource. In China, in efforts to cope with this epidemic, an “Anti-epidemic Expert Group” was launched to formulate quarantine, diagnosis, treatment, and reporting protocols (81). Also, this expert group comprehensively utilized the available professional platforms, including telemedicine system to connect the patients, experts, and information for better managing of the COVID-19 patients not only in China but also around the globe. Additionally, at the time that the WHO declared COVID-19 a pandemic, this expert group invited many Chinese topic experts to use the “Cloud Intensive Care Unit (ICU)” telemedicine platform to share personal experiences in managing critical COVID-19 patients to help mitigate global burden of COVID-19 (81). Simultaneously, based on a “5G Dual Gigabit network,” the West China Hospital of Sichuan University (WCHSU) established a multidisciplinary medical team to deal with cases vulnerable to severe COVID-19, such as the elderly, children, pregnant women, and patients with certain chronic health problems (57) (Figures 5A,B). From January 26 to March 12, 2020, a total of 424 consultations were performed for confirmation of COVID-19 diagnosis (15%), adjustment of antiviral therapy (75%) or respiratory therapy (55%), and management of complications (68%) via this new telemedicine system for severe or critical COVID-19 patients, which has been highly praised by the WHO owing to the increased diagnostic accuracy and improved treatment outcomes for a large rural population in western China. This system can help explain why the case fatality rate (CFR) of COVID-19 cases is only 0.55% in the Sichuan Province of China, much lower than that in Hubei Province (4.64%) and the world averages so far (57).

**Figure 6:**
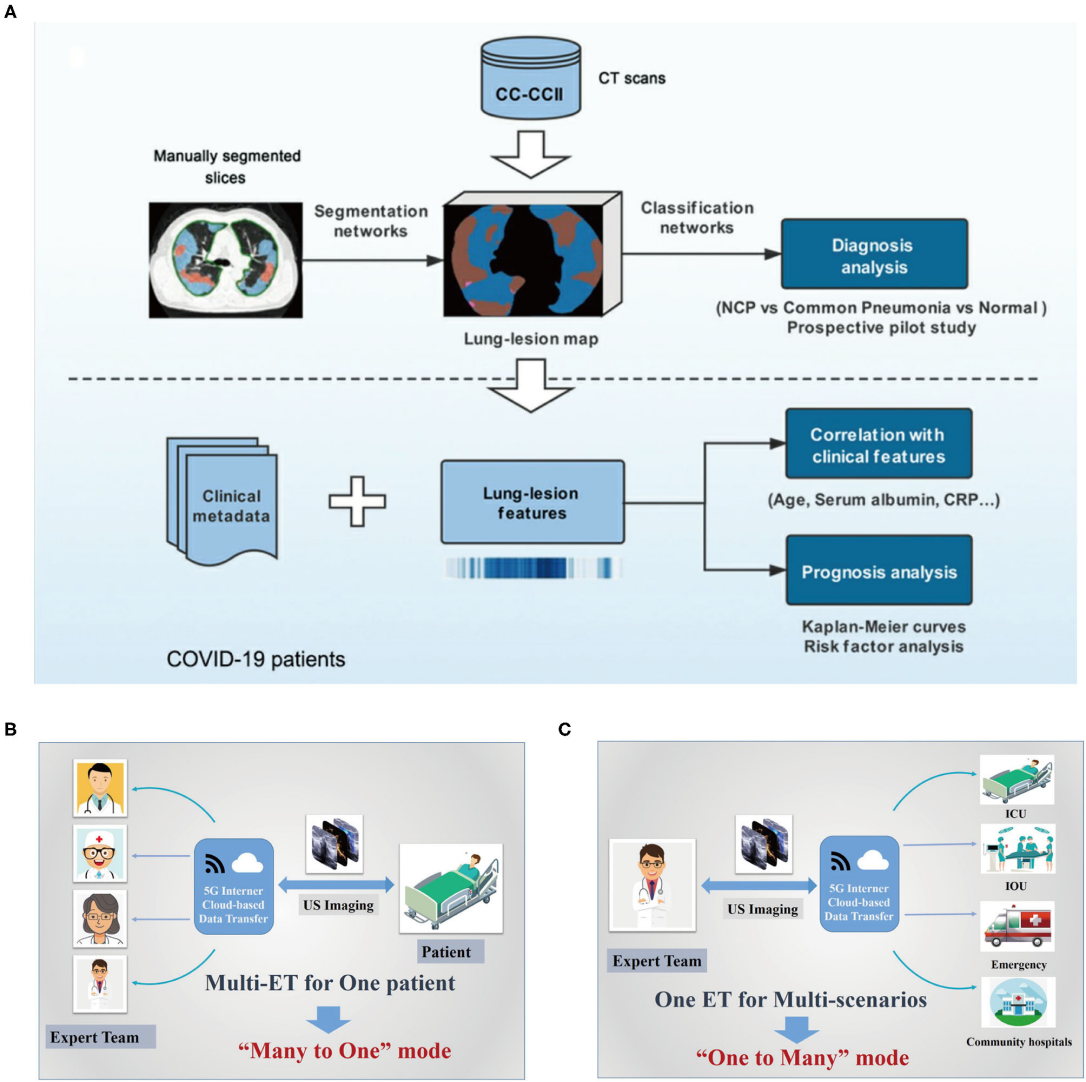
Digital technology-based tele-radiology system used during this COVID-19 outbreak. **(A)** The artificial intelligence (AI) framework for COVID-19 diagnosis and prognosis prediction based on CT imaging (58). **(B,C)** Schematic diagram of the basic structure of tele-ultrasound system based on 5G internet cloud-based data transfer, the “many to one” mode **(A)** and “one to many” mode (59, 60). COVID-19, coronavirus disease 2019; NCP, novel coronavirus pneumonia; CRP, C-reactive protein; CT, computed tomography; 5G, fifth generation; PC, picture archiving and communication system; IOU, intraoperative ultrasound; ICU, intensive care unit.

Driven by the COVID-19 pandemic, various types of medical imaging platforms have been launched worldwide to address the diagnosis problems for COVID-19 patients. Considering that computed tomography (CT) imaging is currently being used for confirming cases of COVID-19 (83, 84), radiologists at WCHSU utilized “5G Dual Gigabit network” to remotely conduct CT scanning on COVID-19 patients, which, to our knowledge, is the first reported case of remote CT scanning during the COVID-19 pandemic (57). Experts at WCHSU were also able to view the CT imaging dataset just as the local clinicians. So far, 152 patients with the aid of telemedicine platform have undergone CT scanning that is remotely guided by WCHSU physicians (57). Also, supported by the telemedicine system and imaging cloud services, radiological medical experts from the Wuhan Union Hospital of China communicated with their Kenyan counterparts regarding the CT imaging diagnosis of COVID-19 and also shared their initial diagnostic criteria for COVID-19 as a way to assist Kenya and other African countries in quickly mastering diagnosis and treatment methods of this disease. And as of March 23, 2020, it reported that 37 counties in Kenya had adopted China's high-end medical imaging equipment, cloud services, and their clinical application training and other one-stop solutions, which were quite helpful for fighting against the epidemic (85). Also, researchers developed AI models that could accurately detect COVID-19 disease and differentiate it from other forms of pneumonia, (61) which can be used remotely by physicians beyond the epidemic areas. As one example, Zhang et al. (58) recently reported their AI-powered CT diagnostic system (Figure 6A) in *Cell*, which could diagnose COVID-19 with an accuracy of 92.49% and had been made available globally to assist the clinicians to combat COVID-19. Abdel-Basset et al. (86) utilized an intelligent framework incorporating emerging technologies (e.g., AI, IoT, big data, autonomous robotics, and 5G) to help make rapid decisions to treat COVID-19 patients while ensuring patient and healthcare team safety.

Additionally, Xu's group (59, 60) launched a “5G-based teleultrasound network” for fighting against this pandemic in China, which incorporated several advanced information technologies including cloud services, AI and robot techniques, greatly facilitating “online” imaging data transmission, and the further “real-time” diagnosis or operation guidance for COVID-19 patients, especially those in ICUs, which also greatly pushes the further development of “many to one” and “one to many” remote clinical patterns (Figures 6B,C). “Many to one” implies that several experts or medical teams synergistically deal with a single clinical scenario, which can integrate expert knowledge from different professional backgrounds, thus facilitating the management of certain critical patients, for example, those in the ICU. Similarly, “one to many” is that one expert or medical team deals with multiple clinical scenarios, which can make better use of the limited expert resources to provide high-quality healthcare for patients, particularly in some extreme environments such as the current pandemic. Importantly, the cloud-based data could further serve as a “Digital Imaging and Communications in Medicine data bank,” providing large-scale shared datasets for the wireless ultrasound (US) imaging analysis, which provides a great opportunity for AI technology development. Synchronously, the “5G remote robotic US diagnostic” system can enable real-time remote control for US scanning, thus eliminating exposure to COVID-19 to the greatest extent. Notably, in Fangcang shelter hospitals, where CT scanning cannot be performed and simultaneously there is a lack of qualified US physicians, it has already been successfully utilized to timely and accurately assess cardiopulmonary function of COVID-19 patients (82) (Figures 5C,D). Together with the reported “remote conduct CT scanning” system, it marks a significant transition of telemedicine from traditional “consultation” mode to a new “practical operation” mode, ensuring excellent US/CT performance even in areas with limited experts or qualified technicians.

The global fight against this pandemic is bound to trigger a great leap in science and technology and thus push for industrial upgrade. One notable example is the rush construction of Huoshenshan Hospital and Leishenshan Hospital in Wuhan, Hubei Province, where in a short span of 2 weeks is equipped with full 5G coverage and several other advanced technologies such as AI, robot, and cloud services. And importantly, the two “smart hospitals” can provide good examples showing the power of digital technology-based telemedicine system in the battle against the novel coronavirus outbreak. For example, an audio and video real-time interconnection system supported by 5G technique has been installed in the two hospitals to facilitate share case information among medical workers within isolation wards. With this system, staff in non-isolation areas and the administrative centers could also see the real-time situation in the isolation area through a large screen at any time and could ensure direct and effective communication at multiple places. In addition, the system can be connected via 5G hot spots, so as to allow multidisciplinary resources from other major cities such as Shanghai, Beijing, and Guangzhou to help remotely diagnose and treat critically ill patients at Leishenshan or Huoshenshan Hospital. This tele-platform runs 24 h a day, so clinicians at the two hospitals could seek help online whenever they have difficulties in case diagnosis or treatment, thus greatly improving treatment levels. Also, robot systems with delivery and disinfection services, as well as remote CT scanning platform, have been adopted in the two hospitals to minimize the potential risk of cross-infection. It reported that, supported by these cutting-edge equipment, during the first 2 months of operation, a total of 2,011 COVID-19 patients were received by the makeshift hospitals and the overall mortality rate was 2.3%, and none of medical personnel contracted this highly contagious disease (87). The two “smart hospitals,” together with Fangcang shelter hospitals, had played an “irreplaceable role” in the battle against COVID-19 in Wuhan of Hubei Province, and China's efficient use of modern technologies has helped the country gain the upper hand in its virus fight, which is a good model for other countries to follow in their fight against this disease.

### Monitoring Status of COVID-19 Patients

During the COVID-19 pandemic, telemedicine services can be applied as a remote-monitoring tool to better understand the evolution of COVID-19 disease, as well as status of high-risk patient populations. At patient level, the evaluation data can be derived from self-reporting system or wearable sensing equipment. An extensive body of literature exist regarding the use of telemedicine system in remote monitoring of some chronic diseases just in this way (88, 89). Indeed, the same concept can also be used for monitoring of infectious diseases such as COVID-19. South Korea, which can be touted as a good global example of effective pandemic response, implemented early a novel “Community Treatment Center” outside hospitals to treat mild COVID-19 patients (90). To minimize the exposure of healthcare providers, patients in the center were asked to self-report their daily temperatures via a phone-based app (inPHR^®^, SoftNet, Seoul, Korea) for clinicians to remotely monitor their disease status, and the adherence rate reached greater than 80%, 2.3% of which were eventually transferred to inpatient units and 31% had been discharged at home (90). Synchronously, considering the importance of reducing the amount of community spread of COVID-19 by keeping people at home, Cleveland Clinic, to our knowledge, is the first reported to implement a home-based intervention program that incorporates a patient-engagement app and monitors symptoms for the early intervention, ultimately promoting a holistic view of healthcare (91). Interestingly, following the widespread interest in remote monitoring for COVID-19 patients, Annis et al. (92) conducted a study to evaluate the outcomes, acceptability, and lessons regarding the remote-monitoring program implementation. An existing third-party application, which previously had been used for enhancing recovery after surgery was introduced for monitoring COVID-19 cases, and 300 patients responded to the satisfaction questions regarding application, of which 74% had been extremely grateful about their experience using this tool, which demonstrated what could be accomplished through shared effective and imperative partnerships between industry and healthcare delivery (92).

Apart from the aforementioned self-reported remote-monitoring systems, robots equipped with devices such as thermometer, cameras, and radar, which could move in isolation areas while controlled remotely by clinicians via mobile phones, have already been used in wards of Fangcang shelter hospitals of Wuhan, Hubei Province, to help protect medical staff members from infection and save protective resources. Also, the automatic infrared thermal imaging temperature detection and warning systems have been adopted in Huoshenshan and Leishenshan hospitals of Wuhan to enable real-time and continuous monitoring of the changes in body temperature and automatically alert the febrile individuals, which could contribute to better management COVID-19 cases via early detection and simultaneously avoid cross-infection. Dong et al. (93) and Alsamhi et al. (94) also summarized the active role of IoT and blockchain during the COVID-19 epidemic, covering the diagnosis of COVID-19 symptoms, quarantine monitoring, and real-time tracking to combat the epidemic. It also revealed the meaningful role of digital technology during the epidemic.

As COVID-19 is an extremely contagious pneumonia that can quickly develop into severe form of respiratory failure, there is a great possibility of overwhelming available ICU resources. The management of critical care patients especially those in ICU has become a topic of great concern. Telemedicine in the ICU provides 24/7 specialist care for critically ill patients combined with remote patient monitoring, which is increasingly touted as a nursing mode to improve efficiencies and quality of care (95). To reduce the contact between healthcare personnel and infected patients, the electronic ICU monitoring and surveillance program such as services offered by Sutter Health, Mercy Virtual Care Center, and Sentara Healthcare enables nurses and clinicians to remotely monitor the status of patients in the ICU (95). At the HIMSS 2020, CLEW, a company focused on intelligent healthcare, demonstrated its AI-powered tele-ICU system to support monitoring of COVID-19 patients' status with certain respiratory deterioration prediction models, which was later installed in two Israeli hospitals (62). Real-time risk stratification will enable timely interventions and thus improve clinical outcomes for critically ill patients.

## Mitigation of the Impact to Healthcare System Indirectly Related to COVID-19

### Telemedicine for Management of Common and Chronic Conditions

Hospitals are potential areas of high COVID-19 cross-infection; thus, to reduce the people accumulation in outpatient centers during this pandemic, online medical consultations, website-based prescription, and delivery services for patients with certain common and chronic diseases have been developed by hospital agencies in many countries. In China, these telemedicine services can be acquired through the websites of hospitals, WeChat accounts, and several widely used apps, for example, “Huayitong” (57). It reported that by March 23, 2020, 31,905 patients had benefited from this service for prescriptions or medicines, greatly reducing the number of patient visits, easing the overcrowding in outpatient clinics, and allaying worry for patients with chronic disease (57).

Actually, telemedicine system is not a new mode for delivering healthcare for common and chronic disease patients, as it has already been used for ongoing management of long-distance clinical care, health administration, and education over the last few years, though several common modalities, such as live-video tele-conferencing, store-and-forward technologies, home monitoring programs, and mobile health applications (96, 97). Despite the great potential of telemedicine system to improve access to healthcare, its uptake is limited in scale due to interstate licensing barriers, inadequate reimbursement, and to some extent the resistance to change and lack of infrastructure (98). The COVID-19 outbreak, particularly together with modern technologies, has, in the short term, accelerated the ever-growing development of telemedicine platform as a distancing measure to cope with this current public health crisis. Integrating the advanced digital technologies into the telemedicine platform makes it rapidly move to be the frontline clinical practice for chronic disease management, due to the demand for prevention and mitigation strategies; it has also been encouraged and facilitated by changes in payer-driven reimbursement policies, as well as government rules and regulations (18). Currently, a variety of telemedicine tools, many of which are free or of low cost, such as Skype, Facebook Messenger video chat, Apple FaceTime, and Google Hangouts video, can be used for consulting specific care (99), and this technology has been re-despoiled and used across various medical specialties including pediatrics, neurology, dermatology, ophthalmology, psychiatry, and oncology (99). For example, Serper et al. (100) in the context of COVID-19 crisis, launched a “telehepatology tertiary-care team” (telemedicine for advanced liver disease) via a VidyoConnect™ between tertiary care site and referring site, to guide the community-based gastroenterology practice. Net Promoter Score (NPS), a gold standard customer satisfaction tool, was calculated by the authors to help evaluate patient likelihood of recommending the telehepatology service to surroundings, and a mean NPS of 92 (range from −100 to 100) was acquired from the respondents, indicating they had undergone a highly satisfied and excellent experience, well above the levels typically seen in healthcare settings, and thus telehepatology was proved to be a feasible, acceptable, efficient way to provide care to these patients with complex liver diseases while not compromising clinical care (100). Additionally, Grossman et al. (18) presented their tele-neurology platform in response to the COVID-19 pandemic by using synchronous audio and video connections between provider and patient, also integrated with an EMR, and showed that it is clinically meaningful mode for virtual services. Similarly, Garg et al. (101) provided a continuous glucose monitoring system by utilizing the commercially available software (i.e., Dexcom, Clarity, and Glooko) to manage type 1 diabetes virtually. Indeed, type 1 diabetes is particularly suited to such a kind of remote care, as most of the visits are built around reviewing data and consulting therapies.

With the WHO declaring this pandemic, there is an inevitable impact on cancer patients; however, due to the limited data, currently, there are no availably international guidelines to enable the management of these patients in times of crisis. In an effort to minimize the interruption of cancer treatment, telemedicine has been recommended as a practical approach by an international collaborative group including eight countries, (102) to support cancer patient management during such an infectious pandemic. Also, Prasad et al. (103), drawing upon their experience, compiled a set of guidelines and a valuable patient handout for head and neck cancer patients to optimize telemedicine visit during the era of COVID-19. Synchronously, Quek's group (104) developed a telemedicine platform (video conferencing) between interventional radiologists and nuclear medicine physicians during this crisis to ensure continuity of care for cancer patients requiring yttrium-90 radioembolization; and at the time of writing their article, three cases including two cases of hepatocellular carcinoma and a metastatic hepatic carcinoma had been successfully treated without complications via this technology. Taiwo et al. (105) also proposed a remote smart home healthcare support system (ShHeS) that can be used by patients who need regular health checkups to receive consultations and doctor's prescriptions at home. Considering the importance of telemedicine system, the European Association of Nuclear Medicine (EANM) and the Society of Nuclear Medicine and Molecular Imaging (SNMMI) promulgated guidelines for the tele-nuclear medicine in remotely interpreting routine nuclear medicine studies, as well as in interpreting emergency studies in on-call settings (106). This outbreak can enable a wider adoption of tele-nuclear medicine during the post COVID-19 era—not only in patient diagnosis and therapy but also in clinician education for certain developing countries with limited access to the formal training in nuclear medicine.

### Tele-Surgery

With the evolution of the COVID-19 outbreak during March 2020, increasing concerns on conserving healthcare resources led to wide calls for delaying or canceling non-urgent services. As such, the Centers for Medicare & Medicaid Services (CMS), on March 18, 2020, announced that all the elective surgeries and non-essential surgical procedures should be delayed. This announcement from CMS came as a specific problem for clinicians, as accurately interpreting the meaning of “elective” and well balancing this definition with patient health can sometimes become a major challenge even for the most experienced surgeons (107). In this context, tele-surgery such as remotely guiding the surgical procedure or robot-assisted remote surgery can be the best option for both doctors and patients.

Remote surgery that is based on the mutual telecommunication information, such as image, video, and audio, digitally transmitting via wireless or cable networks, enables surgeons to manipulate surgical robots to perform operations across distance (108, 109). The instability of network and system delay should be the main obstacles of robot-assisted real-time tele-surgery. Fortunately, the recent revolution of 5G network with spectacular performances in high speed and low latency (78) has facilitated the clinical practice of remote surgery. Tian et al. (63) in collaboration of 5G network and orthopedic robot technology performed 12 cases of tele-robotic spinal surgery (Figures 5E,F). All the patients had substantial relief from their symptoms, while without any intraoperative adverse event. Actually, robot-assisted spinal tele-surgery has been regarded as a popular and reliable surgical technique in the past few years (110–112). Roser et al. (113) reported an accuracy of up to 99% for pedicle screw placement by the robot-assisted spinal surgery. Similarly, Lonjon et al. (114) presented that the accuracy of the pedicle screw placement was 97.3% by using ROSA robot system, higher than that of freehand method (92%). Nowadays, the breakthrough in 5G network system and robot technology, particularly sparked by this health crisis, makes the fast-paced practice of tele-robotic surgery. Huddy et al. (115) reported a simultaneous installation of the da Vinci Si and Versius robotic system, which provided an emergency surgical strategy during a COVID outbreak with excellent outcomes and high patient satisfaction.

### Tele-Psychology

Deep emotional traumas in society overwhelmed by huge human disasters, such as global pandemic diseases, man-made tragedies, natural disasters, social crises, and war conflicts, can cause plenty of stress-related disorders (116). Following this broader context, although treating of COVID-19 patients, virus containment, and vaccine development are the critically urgent issues that should be addressed, it is mandatory to start focusing on the long-term effects on the mental health of the global societies. Even the WHO technical guidance note warned that “the main psychological impact to date is elevated rates of stress or anxiety,” due to the public health crisis and destabilized economic reasons. In the United States, Hamel et al. (117) launched a coronavirus poll and showed that 32% of the adults feel stress and worry associated with coronavirus, which had negative effects on their mental health, with a “major” impact rate of 14%. Also, in China, a COVID-19 poll of 2,091 inhabitants presented that the prevalence of psychological symptoms in mainland China 1 month after the outbreak was 4.6%, while this rate in provinces with higher number of infected cases was 18.4% (118). These scientific papers are important in enabling global health authorities to allocate available health resources and undertake timely actions to minimize the psychosocial impact of this pandemic on the world population. Limited resources of psychosocial services in many countries around the world have been illustrated, which should be further stretched by increasing demand during the global COVID-19 pandemic. Comprehensive approaches based on telepsychiatry is proposed to cope with the lack of access to mental health services, which includes AI, as well as an array of advanced technologies, like internet-based psychological tools and services. For example, online psychological counseling services (for example, WeChat-based resources) currently have been widely developed by mental health professionals from medical institutions or academic societies throughout 31 provinces in mainland China (119). Also, online mental health self-help intervention systems, such as online cognitive behavioral therapies for anxiety, depression, and insomnia, have been established (120). Additionally, several AI programs have already been put into use as interventions for certain psychological crises during this epidemic. For instance, individuals at risk of suicide can be recognized by Tree Holes Rescue (a kind of AI program) (64), by monitoring and analyzing the messages posted on Weibo and further alerting the designated volunteers to take action accordingly. In addition, Di Carlo et al. (121) summarized the important role that technologies such as telepsychiatry played in the management of mental health assistance during the COVID-19 outbreak. The technology offers a promising approach to tele-mental health services.

This COVID-19 outbreak has brought serious psychological impact on societies, while leading to proposals of lots of technology-assisted psychological intervention models based on the internet-based technologies, AI technique, particularly the popularization of 5G mobile networks and smartphones, enabling individuals to acquire telepsychiatry services during this COVID-19 outbreak. All the available tools should be fully utilized as an important part of the whole package of measures to mitigate negative health effects of this current global coronavirus pandemic.

### Tele-Education and Tele-Conferencing

The impact of this rapidly evolving crisis has pervaded nearly the whole society and is now threatening the medical education and conferences. Due to restrictions declared by the Centers for Disease Control and Prevention and other public health organizations, in-person medical education and academic conferences should be suspended to help address the rapid spread of virus, which is a huge challenge for students, the education sector, and medical staffs. Telemedicine platform can provide tele-education and tele-conferencing services, which allow a great deal of flexibility and have been proposed and adopted widely as an effective alternative model. Although this method cannot completely replace the traditional face-to-face form, telemedicine may prove to be an appropriate way to solve the current cancellation of courses and medical activities.

Chick et al. (122) implemented a tele-conference format for their weekday academic conferences by using a commercial online software (GoToMeeting; LogMeIn Inc., Boston, MA, USA), which is free to the users with the paid institutional accounts. For the most part, it is logged in via computers, while this program can also be accessed through tablets or smartphones, thus allowing learner engagement from any place. Similar capabilities are currently accessible from a variety of platforms, such as Skype (Skype Technologies, Palo Alto, CA, USA), WebX WebEx (Cisco Webex, Milpitas, CA, USA), and Zoom (Zoom Video Communications, San Jose, CA, USA). As this strategy is widely successful, the authors call on collaboration between nearby institutions, particularly in a form of educational tele-conference, to ensure trainees have access to high-quality education during difficult times (122). Synchronously, due to the suspended face-to-face teaching in the context of coronavirus, Imperial College London is now using EdTech, an education technology, to keep delivering its mission (123). Also, as an alternative to the clinical placements, Imperial students are being given access to the online recordings of patients, and Imperial clinicians are performing tele-teaching through computers on the hospital sites, which have displayed excellent student interaction. Additionally, some medical schools are now turning to remotely conduct assessments, in order to ensure that graduating students have already met the required competencies just before they begin clinical practice (124).

After a meeting in Massachusetts was related to a number of suspected cases, global medical conferences have been canceled or postponed (125). Many important meetings and symposiums should be held in a virtual way for academic exchange or special case sharing and analysis, which might be the only solution at present. In the United States, Academic Life in Emergency Medicine (ALiEM) launched an ALiEM Connect to demonstrate live stream videos and fully discuss with others in an effort to meet the requirements of weekly conferences (126). Also, in the University of Miami, Miller School of Medicine in collaboration with the Clinical Good Hope in Peru and Universidad Peruana Union developed a video conferencing program to expand epidemic knowledge by introducing different clinical cases for the invited physicians to analyze and diagnose, playing a potentially important role in this pandemic (127).

As health systems are set to be further stretched due to the increasing burden of the COVID-19 pandemic, disruptions in medical education and conference are inevitable across the whole world. However, in the year 2020, there is an ecosystem of universities, healthcare agencies, and associations, as well as charities and education platforms, which can understand the trend toward the tele-technology enhanced learning and academic communication, which might be highly beneficial. Such approaches not only can be utilized for effectively tackling the medical dilemma during this crisis but also will serve to lay the foundation for teaching and academic exchange during future disasters and beyond, which may imply a fast unexpected shift from one form of academic activities and education to another, with wide long-term effects to everyone involved.

## Opportunities and Challenges

**Figure 7:**
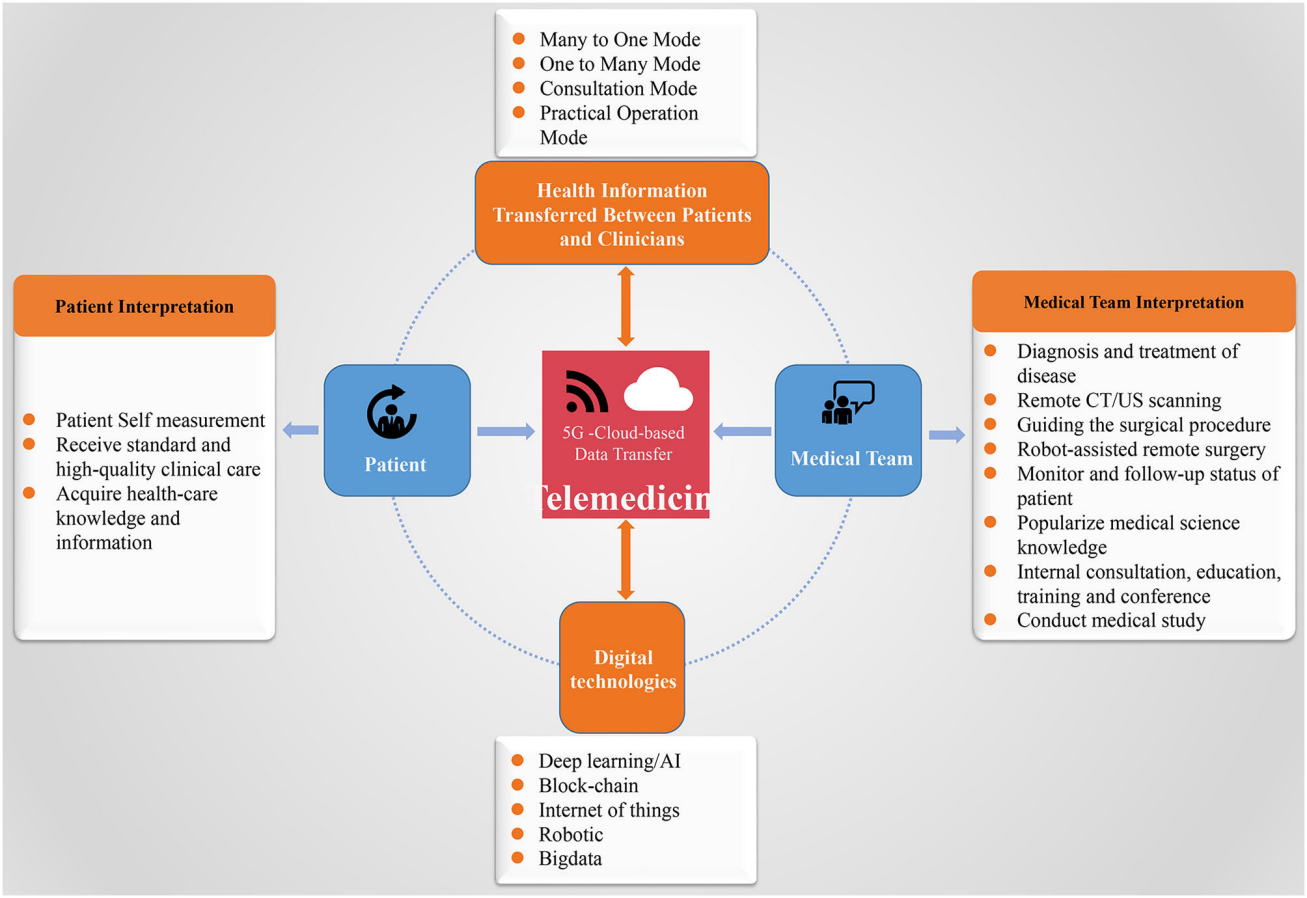
The proposed data flow of telemedicine for clinical care: to maximize clinical care though telemedicine system, a closed loop is quite necessary, which involves healthcare data derived from patients and practitioners; transferred via 5G-Cloud internet; interpreted by the patients, medical practitioners, or with certain automated platforms (e.g., AI, robot, and big data analysis); and returned back to the patients and medical staff for better clinical decisions. And in turn, the large-scale shared cloud-based data would provide a great opportunity for AI development. 5G, fifth generation; AI, artificial intelligence; CT, computed tomography; US, ultrasound.

**Figure 8:**
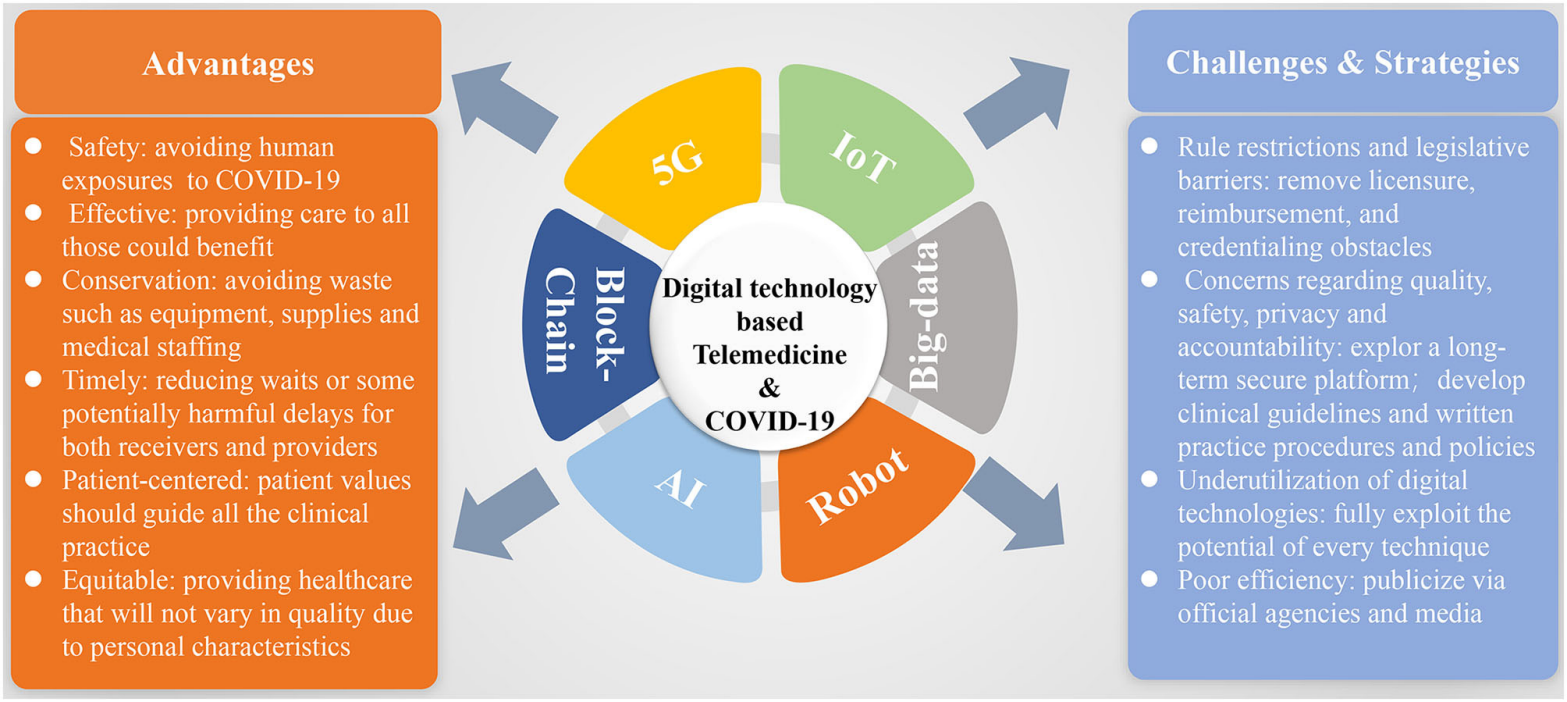
Summative scheme of digital technology-based telemedicine used during the COVID-19 outbreak with its advantages, challenges, and strategies for future wide application. COVID-19, coronavirus disease 2019; 5G, fifth generation; IoT, internet of things; AI, artificial intelligence.

In the context of the COVID-19 outbreak, the rapidly growing modern technologies have brought powerful potential for many applications, which will globally transform the way in which we work, learn, and live. Among them, healthcare is such a specific industry that is experiencing an interesting transformation by integrating these telecommunications technologies. Indeed, to maximize clinical care though the telemedicine system, a closed loop is quite necessary, which involves patient- or practioner-derived healthcare data and internet (e.g., 5G-Cloud)-based data transfer interpreted by the patients, medical practitioners, or certain automated platforms (e.g., AI, robot, and big data analysis) and returned back to the patients and medical staffs for better clinical decisions. And in turn, the large-scale shared cloud-based data would provide a great opportunity for AI development (Figure 7). Increasingly, great amounts of real-world experience regarding digital technology-based telemedicine system have already been developed and can be conducted in four modes (i.e., many to one mode, one to many mode, consultation mode, and practical operation mode) (Figure 7), which increase the medical staffs' capacity to offer high-quality care to more patients while protecting the personal health of medical staffs and saving public resources, by increasing the efficiencies and methods in which expert evaluation could be provided beyond geographic restriction. Importantly, telemedicine system has been demonstrated to be quite useful in handling critically ill patients, as well as the stable outpatients without barriers of time and distance. These are all good examples of ways in which telemedicine based on several digital technologies can currently address these unique challenges posed by an infectious disease outbreak such as COVID-19. Optimizing the application of telemedicine in this pandemic uniquely brings six domains of healthcare quality: as follows (Figure 8): (a) safety: avoiding human exposures (healthcare providers, patients, and exposed community) to COVID-19 and reducing the risk of SARS-CoV-2 infection; (b) effective: providing care to all those who could benefit, for not only critically ill patients acquiring expert services but also people with mild illnesses getting the supportive care to avoid the consequences of delayed diagnosis, as well as reducing their stress and anxiety; (c) conservation: avoiding waste of equipment, supplies, and medical staffs; (d) timely: reducing wait time or some potentially harmful delays for both receivers and providers; (e) patient-centered: providing healthcare that relies on individual patient needs and preferences and ensuring that patient values should guide all the clinical practice; and (f) equitable: providing healthcare that will not vary in quality due to personal characteristics including sex, ethnicity, socioeconomic status, and geographic location, as well as the physician engagement levels. Despite the great potential of telemedicine in clinical application, we should note that its uptake has been variable, mainly due to interstate licensing barriers, inadequate reimbursement and concerns associated with the technical and clinical quality, safety, privacy, and accountability (such as who should be prosecuted if something goes wrong) (98, 128). In this context, several strategies should be adopted to facilitate telemedicine to be globally used and integrated into the public health response to COVID-19 and future outbreaks.

First, the lawmakers and regulatory agencies should promulgate some measures that could facilitate the widespread adoption of telemedicine. The key challenges in wide adoption of telemedicine can be the rule restrictions and legislative barriers. For example, the state licensing restrictions in the United States hamper the providers' ability to deliver healthcare, as they limit the patients' ability to access it. Thus, a national clinical training standard should be set by Committee on Medical Education and be adhered by medical licensing for physicians. Licensure board exams should be national but state-specific. Also, hospital organizations could develop and accept universal credentialing by certain proxy procedures to accelerate the growth of telemedicine delivery, and if possible, an international standard might be developed by the WHO for better transnational cooperation on some difficult public health issues such as COVID-19, because medicine is a special science without boundary. In addition, the full realization of comprehensive implementation of telemedicine requires federal governments and the public and private payers to develop policies that could allow reimbursement of telemedicine services to be equal to that of traditional care to reduce costs. Fortunately, CMS and some private payers have modified the payment policies in response to COVID-19. And Health and Human Services (HHS) waived enforcement of the Health Insurance Portability and Accountability Act (HIPAA) regulation to allow the use of video and audio communication for telemedicine visits (129). This pandemic now serves as a call to action for these groups to work closely with medical experts to remove these licensure, reimbursement, and credentialing obstacles, allowing telemedicine to be fully integrated into our healthcare system.

Second, we should invest time and effort in exploring a long-term secure platform if none is being used now, to define telemedicine frameworks and to develop clinical guidelines and written practice procedures and policies using case scenarios if necessary, for any outbreak at local, national, or global scales. The critical aspect that makes deployment of telemedicine services imperfect and inefficient during the COVID-19 outbreak in some countries such as Italy can be the scarcity of a standard workflow management system or a formal input on telemedicine given by health authorities, which is suitable for integration of telemedicine into clinical practice. Often, a standardized telemedicine protocol and monitoring system technology proposal should exist that are developed by the healthcare facility collaborating with the community site and need to be repeatedly tuned and optimized to ensure that telemedicine system can be operated immediately, safely, and effectively, when suitable.

Third, in times full of danger and uncertainty, we should gather all the resources to help patients and ourselves manage this crisis. The ever-growing technologies including 5G, IoT, AI, blockchain, big data, and robotic technology have markedly affected the progression of telemedicine, and they can be mustered and put into action to strengthen the construction and management of the telemedicine system. In an encouraging sign, all the technologies have already been applied in many respects associated with telemedicine in context of the COVID-19 outbreak and have been proved to be of great practical importance, for example, the “big data” resource in case identification, AI in tele-consultations, standardize screening, and tele-psychology. However, the property of these techniques has not been utilized to the most extent. Take the blockchain technique, for example; it can be fully integrated into the infectious disease reporting systems, enabling data to be automatically and directly reported to the final authority and be avoided passing through intermediary processing, which would greatly improve the efficiency of data transmission regarding the infectious disease outbreaks. Importantly, as any arbitrary editing for the data can be impossible, this blockchain technology will make circumstances of the outbreak be transparent to the public without manipulation (130). In addition, the drone technique, which, to our knowledge has not been used in this epidemic, can be applied as a delivery method to ensure timely diagnosis and patient confidentiality. In the future, we can fully exploit the potential of every technique to help improve performance of telemedicine application.

Finally, given that the lack of certain practical recommendations or a common repository over telemedicine for patients or even some medical staffs can be responsible for the current poor efficiency of care delivered via telemedicine, we should publicize and demonstrate the operation methods of telemedicine through the official agencies and media software, to ensure that both the providers and patients develop a routine way of thinking while performing clinical practice. Also, we can incorporate telemedicine into education and training process to increase routine use of telemedicine services. The preparative work should ensure that the people, equipment, administrative, and systems are all in place to respond immediately and effectively. Such preparedness is of vital important for response. Systems that are not used on a daily basis can rarely work well during disasters; telemedicine is no exception. All stakeholders are encouraged to collaborate to promote the evidence-based use of the telemedicine services during this pandemic and future outbreaks.

## Conclusions

In conclusion, although the world continues to adopt some classic public health measures to cope with the COVID-19 pandemic, there are now various ever-emerging technologies that can be used to facilitate the application of telemedicine system to augment and enhance the traditional public health strategies. The COVID-19 pandemic can make a good “case study” for presenting the potential benefits of telemedicine in real-world clinical practice owing to those advanced technologies. Telemedicine provides a great opportunity to fully take advantage of modern technologies and simultaneously leverage the progressive push toward value and efficiency. Although there is a longer-term goal, the good performance of telemedicine to tackle this enormous, global public health challenge in the year 2020 will undoubtedly create great opportunities to increase the governmental and public acceptance of such a technique in the field of healthcare in the future, as well as to inspire lawmakers and relevant regulatory agencies to promulgate certain measures that will gain more widespread adoption of the telemedicine technology. And so, as predicted by Wootton (131), in the coming years, the term telemedicine will probably lose the “tele-” prefix and become “medicine” with all the telemedicine work regarded as part of routine medical practice.

## Author Contributions

W-WY and H-XX conceived this project. W-WY, H-XX, and LC designed and supervised the project and commented on the project. W-WY, Y-TS, and LC wrote the manuscript. All the authors contributed to the discussion during the whole project.

## Conflict of Interest

The authors declare that the research was conducted in the absence of any commercial or financial relationships that could be construed as a potential conflict of interest.
